# ^68^Ga-labeled WVP peptide as a novel PET probe for molecular biological diagnosis of unstable thoracic aortic aneurysm and early dissection: an animal study

**DOI:** 10.3389/fcvm.2023.1048927

**Published:** 2023-06-12

**Authors:** Xia Lu, Meilin Zhu, Lingzhou Zhao, Feiran Qi, Heng Zou, Peng He, Haizhong Zhou, Kuangyu Shi, Jie Du

**Affiliations:** ^1^ Department of Nuclear Medicine, Northern Jiangsu People’s Hospital, Yangzhou, China; ^2^ School of Basic Medical Sciences, Ningxia Medical University, Yinchuan, China; ^3^ Department of Nuclear Medicine, Shanghai General Hospital, Shanghai Jiao Tong University School of Medicine, Shanghai, China; ^4^ Beijing Anzhen Hospital, Capital Medical University, Beijing, China; ^5^ Beijing Institute of Heart, Lung and Blood Vessel Diseases, Beijing Anzhen Hospital, Capital Medical University, Beijing, China; ^6^ Department of Clinical Medicine, Cellomics (Shenzhen) Co., Ltd, Shenzhen, China; ^7^ Department of Medical Research, Xiangpeng Youkang (Beijing) Technology Co., Ltd, Beijing, China; ^8^ Department of Nuclear Medicine, University of Bern, Bern, Switzerland; ^9^ Department of Informatics, Technical University of Munich, Munich, Germany

**Keywords:** molecular biological diagnosis, type IV collagen detection, WVP peptide, PET/CT imaging, thoracic aortic aneurysm and dissection

## Abstract

**Objective:**

Type IV collagen (Col-IV) is a prospective biomarker for diagnosing and treating of unstable thoracic aortic aneurysm and dissection (TAAD). This study aims to evaluate the feasibility of ^68^Ga-labeled WVP peptide (^68^Ga-DOTA-WVP) as a novel Col-IV-targeted probe for TAAD biological diagnosis using PET/CT.

**Methods:**

WVP peptide was modified with bifunctional chelator DOTA for ^68^Ga radiolabeling. Immunohistochemical staining was used to evaluate the expression and location of Col-IV and elastin in aortas treated with 3-aminopropionitrile fumarate (BAPN) at different time points (0, 2, and 4 weeks). The imaging performance of ^68^Ga-DOTA-WVP was investigated using Micro-PET/CT in a BAPN-induced TAAD mouse model. The relationship between ^68^Ga-DOTA-WVP uptake in aortic lesions and the serum levels of TAAD-related biomarkers including D-dimer, C-reactive protein (CRP), and serum soluble suppression of tumorigenicity−2 (sST2) was also analyzed.

**Results:**

^68^Ga-DOTA-WVP was readily prepared with high radiochemical purity and stability *in vitro*. ^68^Ga-DOTA-WVP Micro-PET/CT could detect Col-IV exposure of unstable aneurysms and early dissection in BAPN-induced TAAD mice, but little ^68^Ga-DOTA-WVP uptake was shown in the control group at each imaging time point. The differences of Col-IV expression and distribution of ^68^Ga-DOTA-WVP both in TAAD and control groups further verified the imaging efficiency of ^68^Ga-DOTA-WVP PET/CT. Additionally, a higher sST2 level was found in the imaging positive (*n* = 14) than the negative (*n* = 8) group (9.60 ± 1.14 vs. 8.44 ± 0.52, *P* = 0.014).

**Conclusion:**

^68^Ga-DOTA-WVP could trace the exposure and abnormal deposition of Col-IV in enlarged and early injured aortas, showing a potential for biological diagnosis, whole-body screening, and progression monitoring of TAAD.

## Introduction

Thoracic aortic aneurysm and dissection (TAAD) is a life-threatening vascular disease, especially when unstable aneurysms with inflammatory conditions progress to aortic dissection, which starts from a tear in the intimal layer of the aorta and bleeding within the media (1, 2). Dissection expands rapidly, leading to serious complications such as rupture or organ ischemia (3). Current clinical guidelines suggest surgical intervention for aortic aneurysm and dissection when the vessel reaches >5–5.5 cm or a growth rate of >0.5 cm/year (4, 5). Unfortunately, this approach oversimplifies complex aortopathy. Up to 50% of ascending thoracic dissections occur in vessels with diameters below the threshold for surgical intervention (6). It has been pointed out that misdiagnosis and delayed management often occurs in clinical workflow even among transfers to aortic referral centers and dramatically worsens the outcomes of patients with TAAD (7, 8). On the other hand, in individuals with thoracic aortic disease, elective endovascular aortic repair and replacement surgery have improved and may be lifesaving; however, these conditions are still associated with increased risk of failure and adverse outcomes (9). Detailed information on the associated findings of the structural and functional biological characteristics are helpful in selecting the best management plan and repeatedly assessing the patient's response to treatment. Therefore, a multidisciplinary approach and earlier and more precise molecular biological diagnosis of unstable aneurysms are imperative to screen the high-risk patients who can benefit from appropriate surgical timing and correct surgical treatment strategy in clinical scenarios to optimize outcomes of TAAD patients.

Molecular imaging, such as hybrid positron emission tomography (PET)/computed tomography (CT), as an adjunctive tool to conventional structural imaging technology, can be used to non-invasively assess anatomic, hemodynamic, and molecular biological features of the aorta, providing a more accurate selection of patients who can benefit from preventative surgical intervention and different options of surgery (10). The complexity of aortic disease is more fully revealed with new functional imaging techniques than with conventional anatomic analysis alone by using a suitable probe to personalize a surveillance regimen or define a more precise intervention threshold to prevent aortic complications (11, 12).

It has been demonstrated that progressive endothelial injury occurs before intimal tearing, including endothelial cell (EC) loss, increased permeability, and subsequent exposure of the subendothelial basement membrane (11, 13). Type IV collagen (Col-IV), a major component of the subendothelial basement membrane, is initially exposed at the sites of EC loss and vessel injury. Patients with TAAD exhibit significantly increased exposure of aortic collagen into the arterial lumen, which may present a novel target for molecular imaging and therapy (14). In our previous studies, a multimodal Col-IV-DOTA-Gd-rhodamine targeted Col-IV by peptide WVP (KLWVLPK) probe was designed to identify the exposed Col-IV in the degenerated aorta for early detection of TAAD via magnetic resonance imaging (MRI) and monitor disease progression in TAAD (15). We also reported a multifunctional nanosystem for delivery (TP-Gd/miRNA-Col-IV) that targets the exposed Col-IV by peptide WVP for nucleic acid therapy to treat TAAD and found such targeted therapy could stabilize the vascular structures, preventing the deterioration of TAAD (16). However, there is still a lack of research on radionuclide-based probes for Col-IV imaging that would be more sensitive to monitor TAAD progression than MRI owing to priority of molecular functional detection and whole-body imaging. Thus, the capability for making a biological diagnosis of TAAD remains to be assessed.

Herein, the Col-IV-targeted WVP peptide was radiolabeled with ^68^Ga (^68^Ga-DOTA-WVP) as a novel PET probe for TAAD imaging. This study aims to evaluate the feasibility of ^68^Ga-DOTA-WVP as a Col-IV-targeted probe for PET/CT of unstable thoracic aneurysms and early TAAD biological diagnosis. To the best of our knowledge, this is the first example regarding the development of a WVP-based PET probe for TAAD imaging.

## Materials and methods

### Materials

WVP peptide was manufactured using the solid-phase peptide synthesis method by ChinaPeptides Co., Ltd. (Shanghai, China). During the synthesis process, the C-terminus of WVP was modified with DOTA to obtain DOTA-WVP. Sodium acetate (NaOAc), hydrogen chloride (HCl), 3-aminopropionitrile fumarate salt (BAPN), Sirius red, and picric acid were supplied by Sigma-Aldrich (St. Louis, MO, USA). Rabbit anti-mouse Col-IV and elastin were purchased from Abcam, Inc. (Cambridge, UK). Other chemicals and solvents were supplied by Sinopharm Chemical Reagent Co., Ltd. (Shanghai, China).

### Preparation of ^68^Ga-DOTA-WVP and quality control

^68^Ga was eluted from a ^68^Ge/^68^Ga generator (ITG, Baden-Württemberg, Germany) with 4 ml of 0.5 M HCl solution and collected in a 10 ml sterile vial. One milliliter of eluted ^68^GaCl_3_ was mixed with 65 μl of DOTA-WVP (1 mg/mL) dissolved in 1 M NaOAc. Then the reaction mixture was incubated at 95°C for 15 min. After being cooled to room temperature, the formed ^68^Ga-DOTA-WVP was analyzed using the Agilent 1260 high-performance liquid chromatography (HPLC) system (Agilent Technologies, Santa Clara, CA, USA) equipped with a UV-Vis detector (*λ* = 220 nm) and radioactive flow detector (BioScan, Poway, CA, USA). The SunFire C18 column (5 μm, 4.6 × 250 mm, Waters, Osaka, Japan) was used at a flow rate of 1 ml/min using the following gradient method: 0.1% trifluoroacetic acid in water and acetonitrile (CH_3_CN) (0–20 min, 15%–45% CH_3_CN). The final product was diluted with 0.9% saline and filtered through a 0.22 μm Millipore filter. To assess *in vitro* stability, 500 μl of ^68^Ga-DOTA-WVP (4 mCi) was mixed with 500 μl of phosphate-buffered saline (PBS, 0.1 M, pH = 7.4) at room temperature. The radiochemical purities (RCPs) were tested using HPLC at different time intervals (1, 2, and 3 h).

### Animal experiment and experimental model of TAAD

All animal experiments complied with the Guidelines for the Care and Use of Research Animals established by the ethical committee of Shanghai General Hospital. C57BL/6J male mice were purchased from the Shanghai Laboratory Animal Center of the Chinese Academy of Sciences (Shanghai, China). Three-week-old male mice (weight: 8–12 g) were fed a normal diet (control) or 0.1% Wt/Vol BAPN in drinking water for 6 weeks to establish the TAAD disease model. The lower dose of BAPN used in this study to extend the survival of the mice to monitor the whole process of TAAD formation and evaluated the efficiency of ^68^Ga-DOTA-WVP for early detection of unstable aortic aneurysm and diagnose early dissection (17, 18). Animals grouped TAAD (*n* = 62) and control (*n* = 10) performed PET/CT imaging and immunohistochemistry staining in different pathogenesis to compare ^68^Ga-DOTA-WVP uptake in aorta and expression of Col-IV on aortic lesions.

### PET procedures

The ^68^Ga-DOTA-WVP solution (100 μl, 200 *μ*Ci) was administered as an intravenous bolus to mice with TAAD and the control mice. PET images were acquired at 0.5, 1, and 2 h after injection using a Micro-PET/CT scanner (Siemens Inveon PET/CT scanner, Siemens Healthineers, Erlangen, Germany). All PET studies were reconstructed as a series of three-dimensional (3D) PET images using a two-dimensional-ordered subsets expectation maximization algorithm (four iterations, six subsets), resulting in a voxel size of 0.86 × 0.86 × 0.79 mm. Whole-body CT was used for attenuation correction, and PET studies were corrected for random coincidences, dead time, scatter, and decay.

### Image analysis

PMOD software 3.8 (PMOD Technologies, Ltd., Zurich, Switzerland) was used to perform image analysis. The CT and PET images were co-registered using the “Fuse it” toolkit. Abnormal findings that visually matched the characteristics of TAAD were outlined in transverse slices and automatically adapted to 3D volume. Volumetric regions of interest (VROIs) were placed on the anatomical CT images to identify the thoracic aorta and drawn around the high uptake area within the aortic wall lesions. Additional VROIs were drawn in other important organs, including the liver, muscle, heart, brain, bone, lung, and intestine, to measure the *in vivo* biodistribution of the novel peptide probe. The VROIs in the control group were created in the same manner.

The ^68^Ga-DOTA-WVP uptake was analyzed in the VROI with respect to the maximum standardized uptake value (SUV_max_). To determine the probe uptake in the early and late phases (static) in the defined TAAD regions, data were compared and analyzed at three time points (0.5, 1, and 2 h) in the TAAD group to explore the *in vivo* dynamic information of the novel peptide probe.

### Immunohistochemistry

After imaging, the aortas of mice were excised, embedded in an optimal cutting temperature compound, sectioned, and stained for immunohistochemistry using rabbit anti-mouse Col-IV and elastin antibodies and the secondary antibody was goat antirabbit IgG. Elastin on sections of the aorta in the control mice and mice with TAAD was incubated for 5 min in Lugol's iodine solution, washed twice with PBS, and then incubated with sodium thiosulfate for 5 min. Next, sections were washed for 5 min with running tap water, followed by 70% ethanol, incubated with aldehyde-fuchsin for 10 min, washed with 70% ethanol until they no longer bleached, and stained with Acid Orange G for 10 s.

Collagen was stained directly with Sirius red and saturated aqueous picric acid (1.3% in water). Nuclei were stained with Weigert's hematoxylin for 8 min, washed with running tap water for 10 min, stained with picric-Sirius red for 1 h, and then washed in two changes of acidified water. This achieved near-equilibrium staining that did not increase with longer staining times as shorter times were not sufficient, even when the colors appeared adequate. The slides were then dehydrated in three changes of 100% ethanol, cleared in xylene, and mounted in a resinous medium. The results were observed using a digital slide scanner (3DHISTECH. Ltd).

### Enzyme-linked immunosorbent assay analysis of blood

All collected blood samples were divided into positive (SUV_TAAD_ ≥ 1.6 SUV_liver_) and negative (SUV_TAAD_ < 1.6 SUV_liver_) imaging groups based on the data from ^68^Ga-DOTA-WVP PET/CT imaging. Blood samples were collected in anticoagulant tubes and centrifuged for 10 min. The supernatant was used to measure the levels of serum D-dimer, C-reactive protein (CRP), and serum soluble suppression of tumorigenicity-2 (sST2) using enzyme-linked immunosorbent assay.

### Statistical analysis

Data were expressed as mean ± standard deviation. The differences between the two groups were analyzed using unpaired Student's t-tests, and comparisons between more than two groups were conducted using one-way analysis of variance, followed by Bonferroni's *post hoc* test. For normally distributed data, we used Pearson's correlation test, and we used Spearman's correlation test for data with skewed distribution. Statistical analysis was performed using SPSS 24.0 (IBM Corp., Armonk, NY, USA). *P*-values < 0.05 were considered statistically significant.

## Results

### Radiochemistry

**Figure 1:**
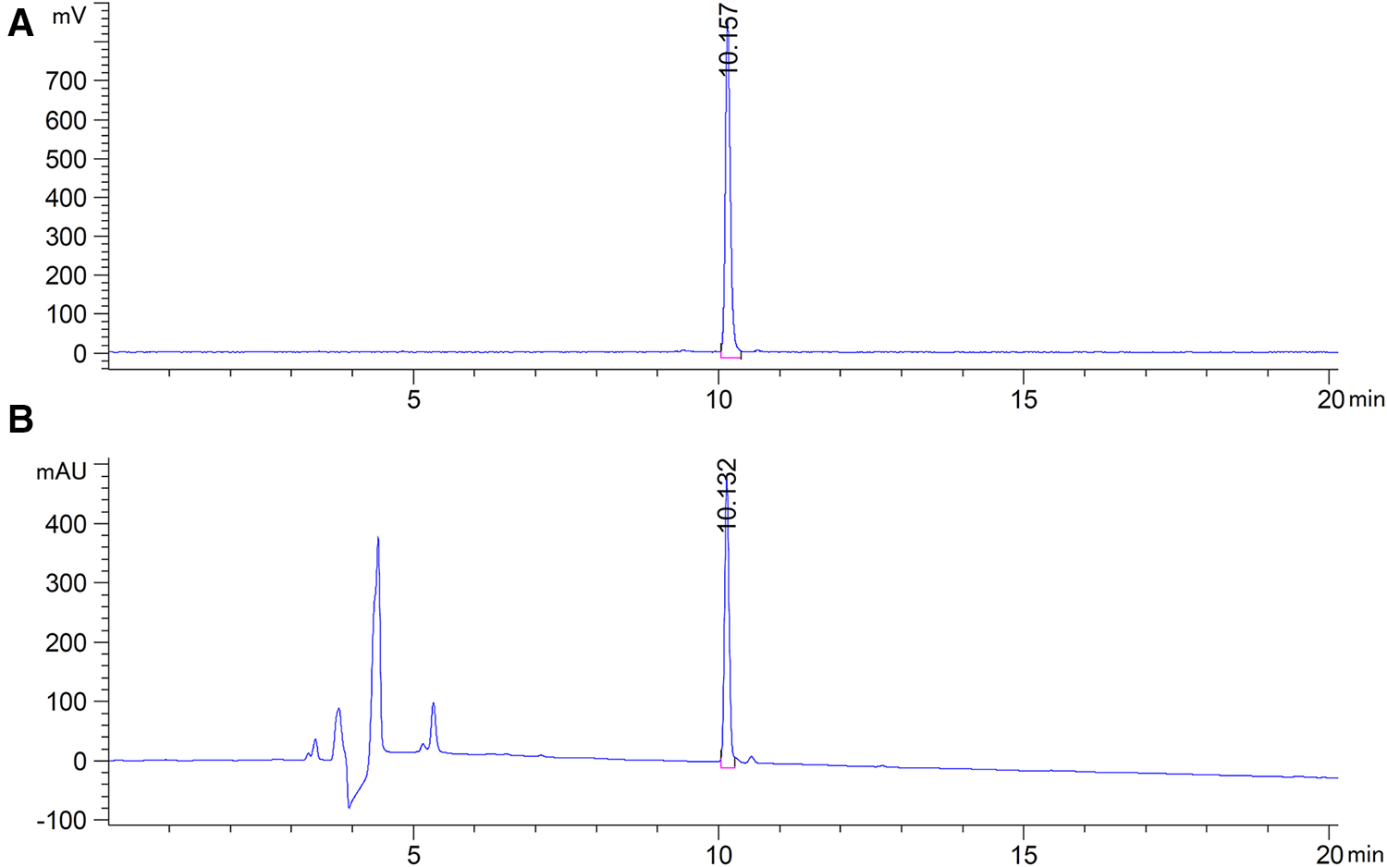
HPLC results of ^68^Ga-DOTA-WVP. (**A**) The radioactive peak shows the position of ^68^Ga-DOTA-WVP. (**B**) The UV peak shows the position of DOTA-WVP peptide, which is consistent with the ^68^Ga-DOTA-WVP, indicating that the precursor was successfully radiolabeled with ^68^Ga.

The WVP peptide was effectively radiolabeled with ^68^Ga via the bifunctional chelator DOTA in 15 min. As shown in Figures 1A,B, the HPLC results of ^68^Ga-DOTA-WVP had a single radioactive peak with a retention time of 10.16 min, which was consistent with that of the corresponding DOTA-WVP (10.13 min). The RCP of ^68^Ga-DOTA-WVP was calculated to be more than 99% without further purification. No obvious changes in the labeling peptide were found in PBS at room temperature within 3 h, suggesting good stability of ^68^Ga-DOTA-WVP *in vitro*.

### Exposure of Col-IV at the site of EC loss in the early stage of TAAD

**Figure 2:**
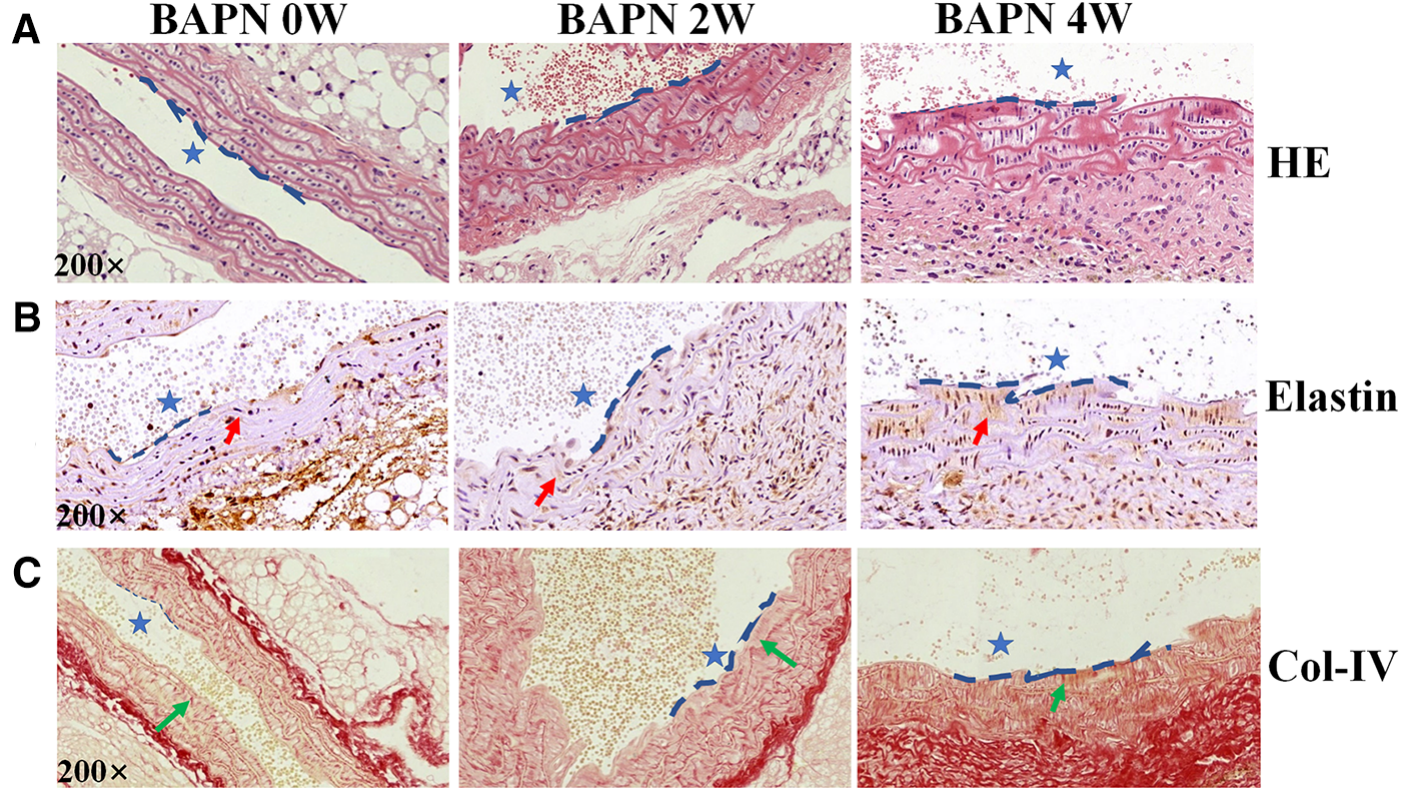
Pathological features of TAAD at different stages (0, 2 and 4 weeks). Mice were fed 0.1% BAPN for 4 weeks to simulate the different stages of TAAD development. (**A**) H&E staining on the aortic arch. (**B**) Elastin staining on the aortic arch (red arrow). (**C**) Sirius red staining of collagen on the aortic arch (green arrow indicates Collagen). The scale bars on the three panels are 50 μm. The blue dashed line is the intima, indicating the vascular lumen (blue asterisk indicates lumen of aorta).

Three-week-old C57BL/6J mice (*n* = 12, weight: 8–10 g) were fed 0.1% Wt/Vol BAPN in drinking water for 4 weeks and observed EC loss occurred as early as the first week of BAPN administration by Evans blue staining, and the area of EC loss increased with progressive TAAD development, as reported in a previous study (15). Elastin staining revealed slight disordering and disruption of elastic fibers that could be detected in the aortic arch after 2 weeks of BAPN administration (Figures 2A,B). Col-IV located under the aortal intima was exposed and gradually increased with BAPN administration during the development and worsening of TAAD from 2 weeks to 4 weeks confirmed using Sirius red staining (Figures 2A,C). These results demonstrated that exposure of Col-IV is a crucial characteristic of early stage of TAAD and high-risk TAAD.

### Micro-PET/CT imaging and biodistribution on early stage of TAAD

**Figure 3:**
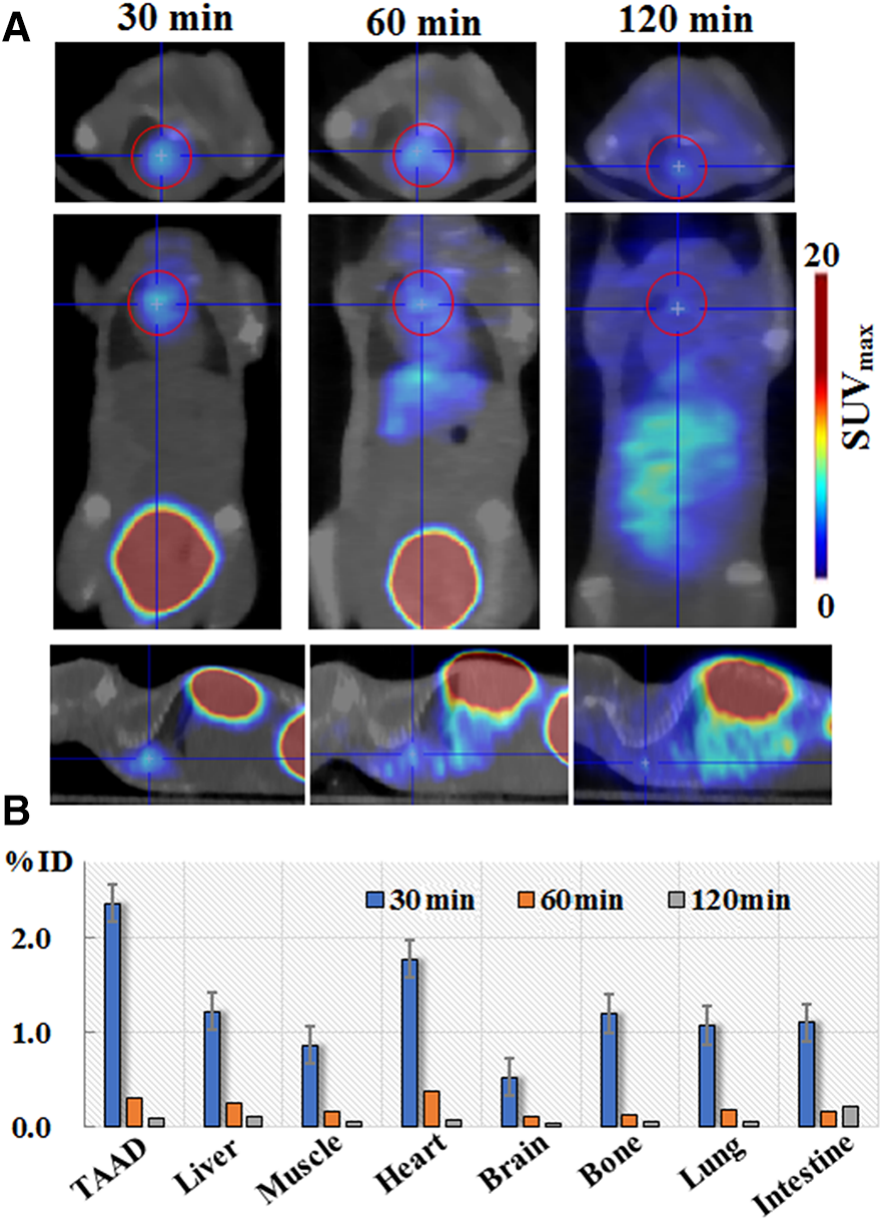
(**A**) Micro-PET/CT imaging and (**B**) biodistribution of ^68^Ga-DOTA-WVP in mice with early stage of TAAD. The thoracic aorta and other tissues of interest were analyzed to determine the uptake levels of ^68^Ga-DOTA-WVP. Data are expressed as SUV_max_ and the percentage of injection dose (% ID).

Following BAPN administration for 2 weeks (early stage of TAAD), the mice (*n* = 12) were intravenously injected with 100 μl of the ^68^Ga-DOTA-WVP solution [(^68^Ga) = 2 mCi/ml] and PET/CT imaging was performed at 0.5, 1, and 2 h after injection. Significantly increased uptake was observed in the thoracic aortic region, followed by the heart (Figure 3). The thoracic aortic lesions were visualized by PET/CT imaging at 0.5 h after injection and signals decreased fast at 1 h and 2 h imaging after injection ^68^Ga-DOTA-WVP owning to good wash-out dynamics *in vivo*. ^68^Ga-DOTA-WVP was excreted through the urinary system; thus, the kidneys and bladder retained abundant tracers. The *in vivo* biological distribution analysis verified the spatial distribution of the images within the analyzed tissues. The ^68^Ga-DOTA-WVP peptide probe focused on injured aortic lesions and had low background signals. In addition, the brain exhibited barely tracer uptake, and the intestine exhibited little tracer uptake, which is important to facilitate accurate diagnosis and reduce radiation dose.

### Molecular biological detection of TAAD by serial PET/CT imaging

**Figure 4:**
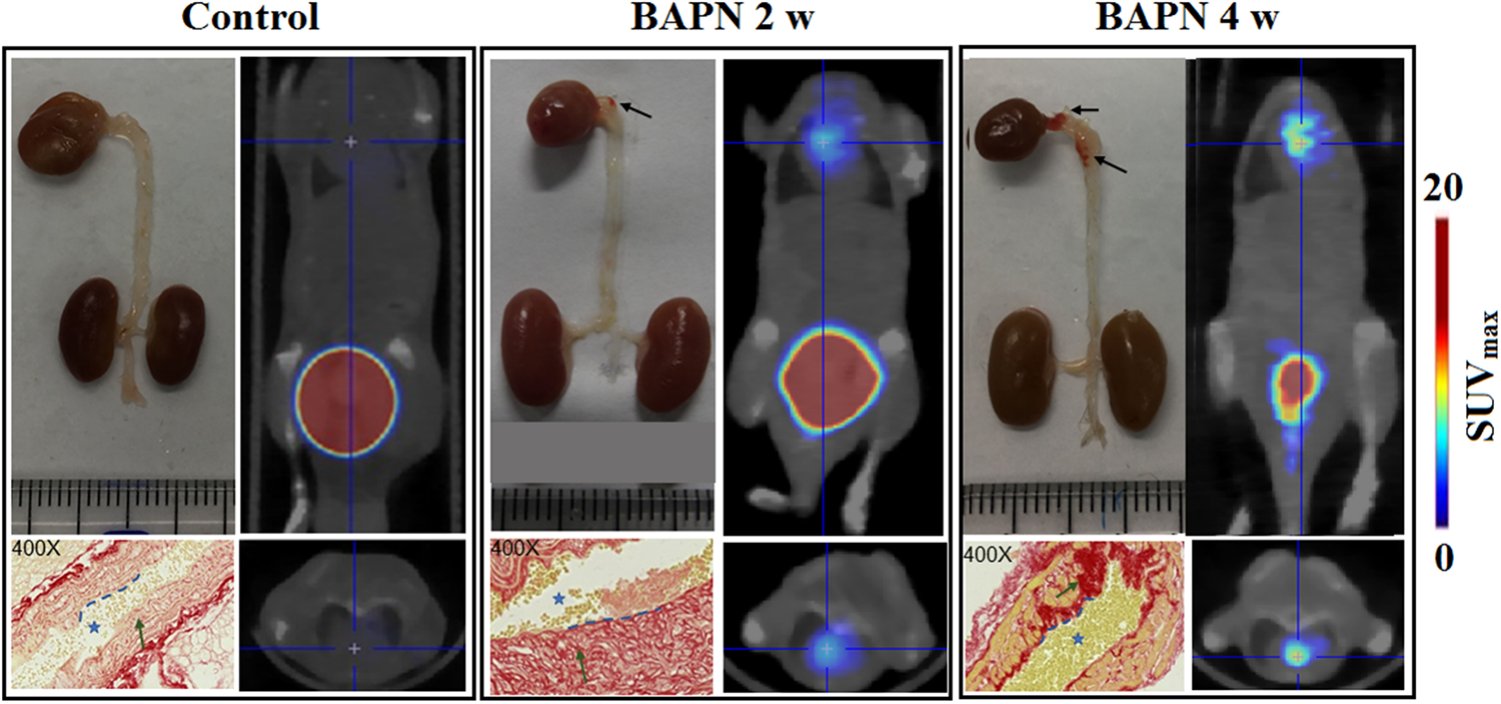
^68^Ga-DOTA-WVP PET/CT images of mice with TAAD in different stages and control mice. The *in vivo* images were verified by photographs of macroscopic features of the thoracic aorta and histological staining (blue asterisk = lumen of aorta; blue dashed line = intima; green arrow = collagen expression; black arrow = TAAD lesions).

We evaluated the efficacy of ^68^Ga-DOTA-WVP in detecting unstable TAAD lesions using PET/CT (TTAD group: *n* = 20 and control group *n* = 10). The aortic diameters of thoracic aneurysm become progressively enlarged with prolonged BAPN administration shown by representative photographs thoracic aorta. Whole-body static images were compared at 0.5 h after injection of ^68^Ga-DOTA-WVP in mice with TAAD and control mice group in different pathologic stage (Figure 4). The thoracic aortic wall with Col-IV abnormal deposition and exposure presented markedly higher uptake of the peptide probe in the TAAD group as early as 2 weeks compared with that in the control group, which was consistent with the results of Elastin and Col-IV staining. Additionally, the ^68^Ga-DOTA-WVP uptake in thoracic aortic lesions gradually increased with the longed BAPN administration, which could be visualized clearly at 4 weeks on serial PET/CT images and accompanied by TAAD progression and marked by more exposure of Col-IV. This was also in agreement with the immunohistochemical staining results (Figure 4). The retention of ^68^Ga-DOTA-WVP in kidneys was significantly higher in the TAAD group than in the control group at 0.5 h after injection (11.53 ± 6.56 vs. 1.21 ± 0.35, *P *< 0.001). Moreover, the ^68^Ga-DOTA-WVP uptake in the heart was also significantly increased in the TAAD group compared to that in the control group at 0.5 h after injection (1.74 ± 1.23 vs. 0.19 ± 0.08, *P *< 0.001).

### Predictive value of ^68^Ga-DOTA WVP uptake for assessing the severity of TAAD

**Figure 5:**
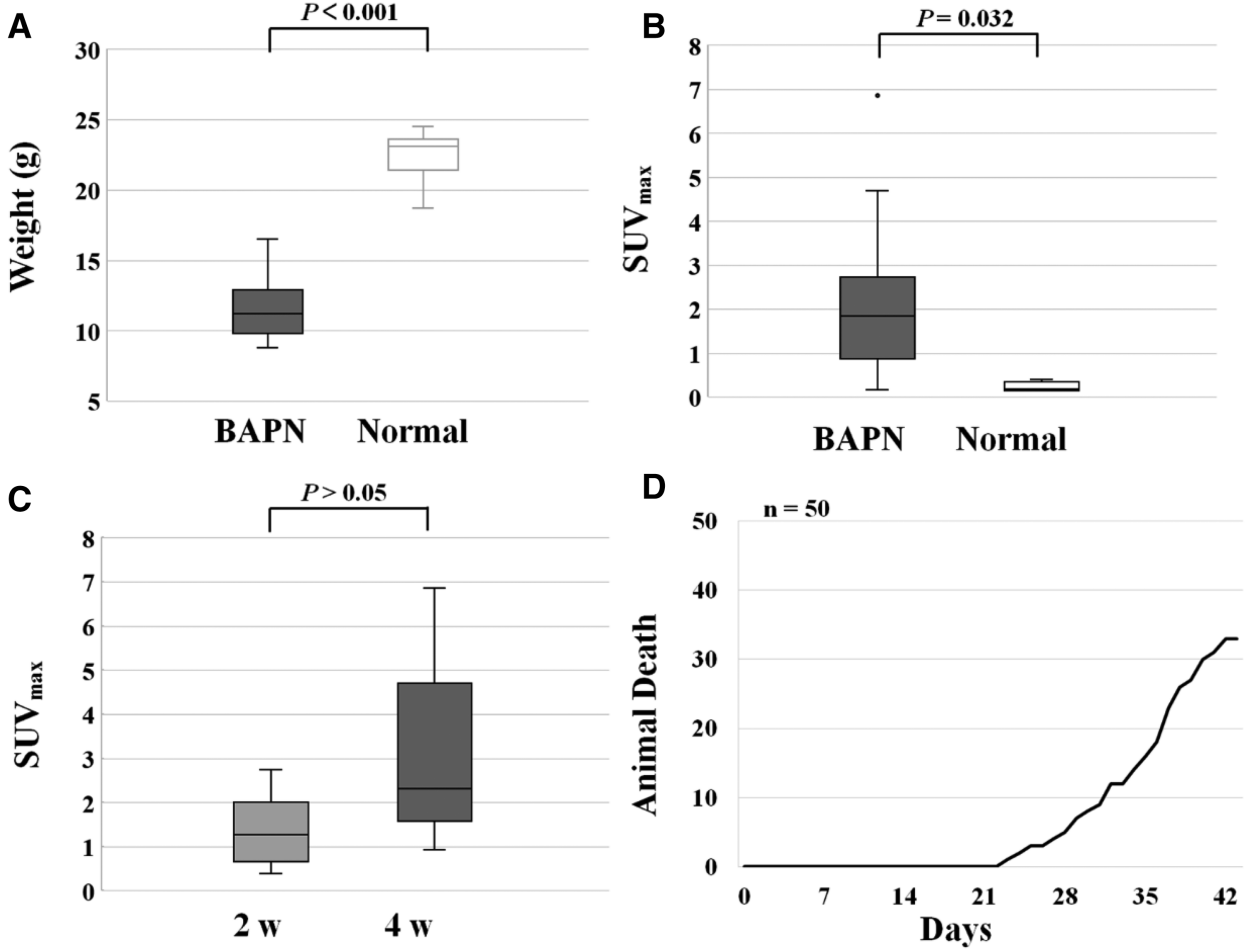
(**A**) Changes in body weight of mice control and TAAD treated mice. (**B**) Increased uptake of ^68^Ga-DOTA-WVP in mice with TAAD in thoracic aortic lesions. (**C**) The comparison of ^68^Ga-DOTA-WVP uptake in thoracic aortic lesions between 2 and 4 weeks after BAPN treatment. (**D**) Ruptures in mice with progressive TAAD (*n* = 50).

The weight of the TAAD mice decreased significantly compared to the normal ones (11.60 ± 2.18 vs. 22.31 ± 1.91, *P* < 0.001) (Figure 5A). In contrast, the ^68^Ga-DOTA-WVP uptake in thoracic aortic lesions increased significantly in TAAD mice (2.14 ± 1.64 vs. 0.23 ± 0.11, *P* < 0.001) (Figure 5B). The uptake value of ^68^Ga-DOTA-WVP in thoracic aortic lesions showed an obvious increase with the progression of TAAD after BAPN administration using serial PET/CT imaging (Figure 5C). The mice did not die from rupture of TAAD until 3 weeks of BAPN administration. The first dead TAAD mouse died from rupture was found 23 days after BAPN administration and gradually increased until 66% (33/50) of TAAD mice died from rupture at 6 weeks after BAPN administration (Figure 5D).

**Figure 6:**
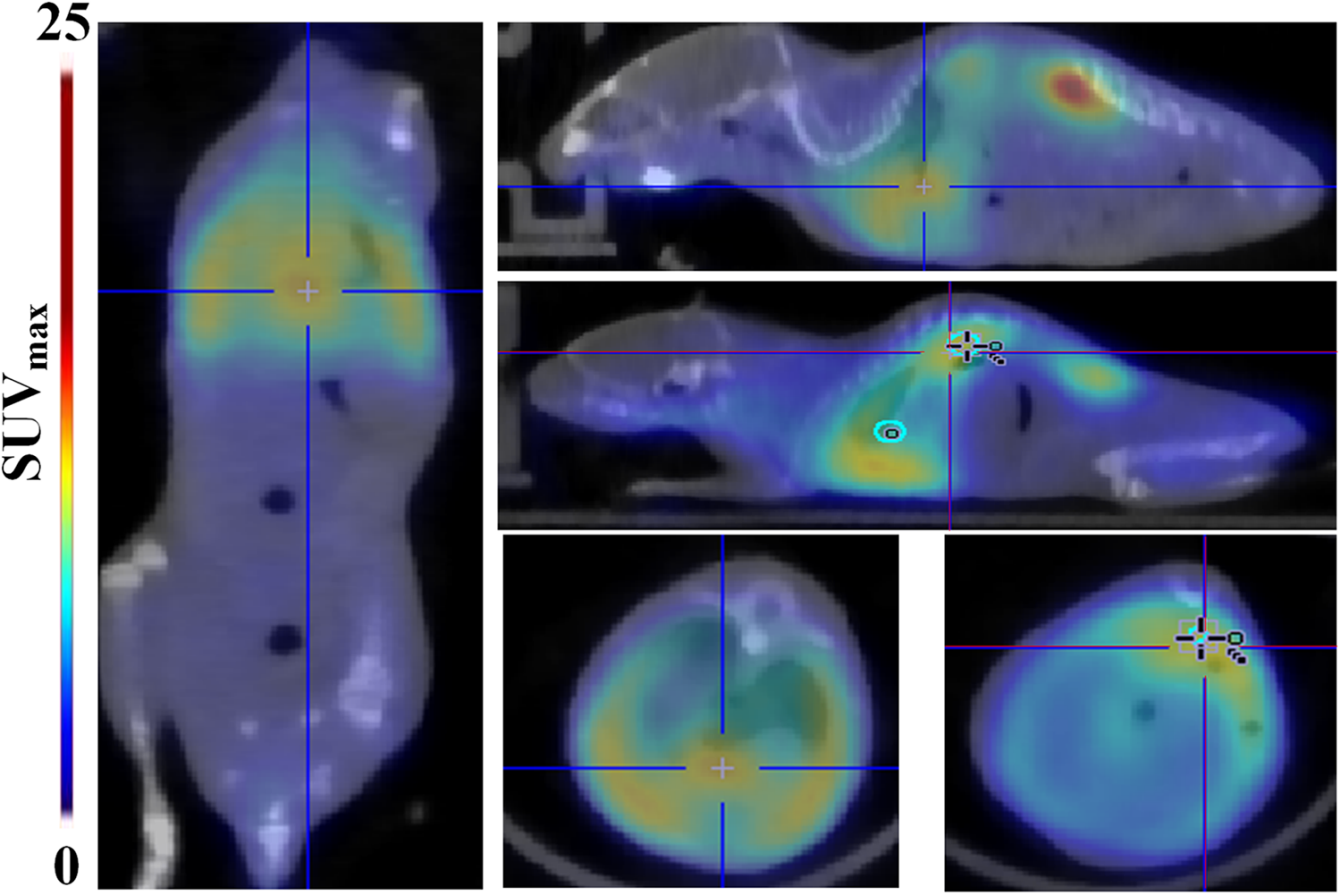
A case of rupture among the TAAD mice. Whole-body ^68^Ga-DOTA-WVP PET/CT images showed significant high uptake of the probe in the aorta, heart, and lung, indicating the large extent and severity of vascular damage caused by BAPN administration. Thoracic aortic dissection is showed in the blue cross and abdominal aortic dissection is shown in the blue cross.

We also present a case report of a TAAD mouse dying from rupture which indicates the predictive value of ^68^Ga-DOTA-WVP PET/CT imaging when assessing the severity of vascular damage after BANP administration. The body weight of this mouse increased slowly and was lighter than other mice in group 2 from 2 to 4 weeks post BAPN administration. When imaged at 4 weeks, the mouse had the lowest body weight among all surviving TAAD mice and was subsequently euthanized by overexposure to isoflurane and found blood clot in chest cavity after dissected. Whole-body images showed a significantly intense concentration of probe was detected in the thoracic and abdominal aorta, heart, and lung. This possibly demonstrated whole body vasculature damage from BAPN administration (Figure 6).

### Relationship between ^68^Ga-DOTA-WVP uptake and serum biomarkers

**Figure 7:**
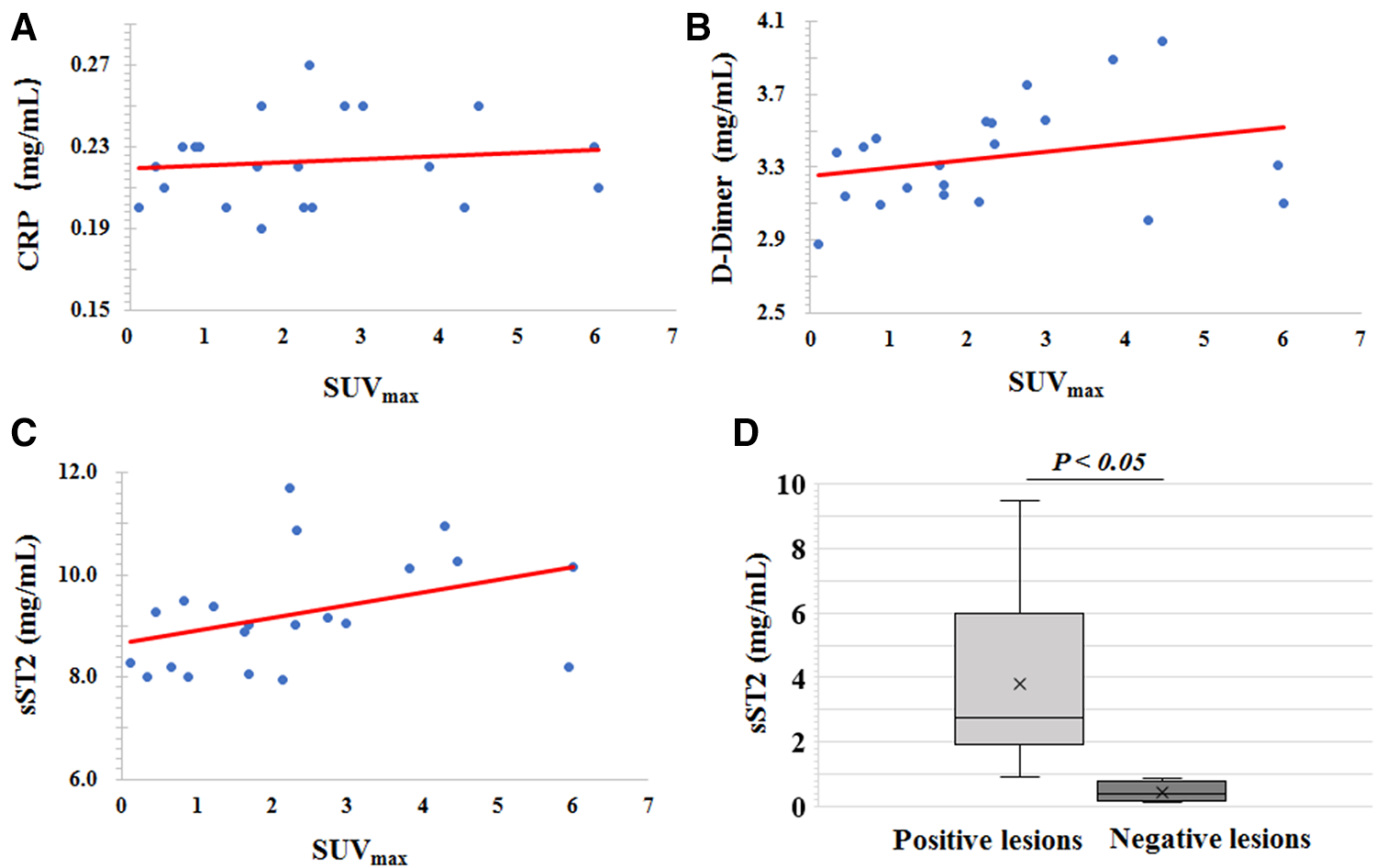
Scatter diagrams of the association analysis of ^68^Ga-DOTA-WVP uptake on thoracic aortic wall and serum levels of (**A**) CRP, (**B**) D-dimer, and (**C**) sST2. (**D**) Comparison of the serum level of sST2 between the positive and negative imaging groups.

The relationship between ^68^Ga-DOTA-WVP uptake on the thoracic aortic wall and serum levels of CRP, D-dimer, and sST2 were analyzed. There was no significant association between ^68^Ga-DOTA-WVP accumulation and serum levels of those biomarkers. We then compared the levels of these biomarkers between the ^68^Ga-DOTA-WVP PET/CT positive (*n* = 14) and negative imaging groups (*n* = 8). The sST2 level was significantly higher in the positive imaging group than in the negative group (9.60 ± 1.14 vs. 8.44 ± 0.54, *P* = 0.014); however, the levels of CRP and D-dimer did not differ significantly between the groups (Figure 7).

## Discussion

In the present study, the novel probe ^68^Ga-DOAT-WVP was designed and successfully prepared for PET imaging, and we found that ^68^Ga-DOAT-WVP PET/CT imaging could extensively detect unstable aneurysms and enable early diagnosis of TAAD (after 2 weeks of BAPN administration) by targeting Col-IV, which was exposed to a small tear site of the intimal aorta. During TAAD development, Col-IV exposure gradually increased with BAPN administration and processive TAAD, owing to the compensatory repair of the degenerated aorta, which was consistent with the gradual increase in ^68^Ga-DOTA-WVP uptake and intensive signal on thoracic aortic wall lesions *in vivo* PET/CT imaging. Importantly, the *in vivo* biodistribution results showed high ^68^Ga-DOTA-WVP uptake in the heart, but lower uptake in the liver, brain, lung, bone, muscle, and intestine, which was beneficial to acquire good imaging quality and have robust potential applications in clinical screening of high risk TAAD patients.

The accuracy of diagnosing unstable aneurysms and early TAAD has varied in each trial, and the appropriate imaging technique to apply in each at-risk population remains unclear. Molecular imaging techniques, such as PET/CT, have shown promise in detecting vessel wall instability at an early stage (19, 20). Our results suggest that ^68^Ga-DOTA-WVP PET/CT imaging could provide whole-body molecular information on Col-IV exposure and expression as an ideal biological characteristic target of unstable TAAD lesions. We showed firstly that ^68^Ga-DOTA-WVP administration resulted in a clear and significantly high uptake in aortic lesions in whole body imaging, which was confirmed by the high SUV_max_ after 2 and 4 weeks following BAPN administration, whereas no obvious uptake was observed in the control group at the same time points. Patients with TAAD reportedly exhibit significantly increased expression of collagen, which may be a new target molecule in the molecular imaging of TAAD (21). This finding concurs with the current study's finding. In the early stages of aneurysm, the basement membrane components that contain collagen increased in the thickened area, and the basement membrane becomes thin from the degeneration of elastin and depletion of smooth muscle cells in the later stages. Smooth muscle cells in unstable aortic aneurysms and dissected aortic media exhibit phenotypic switching from the contractile to synthetic type. Synthetic smooth muscle cells increase collagen synthesis and matrix metalloproteinase-2 production, both of which can promote abnormal collagen deposition and elastin degradation in TAAD and support the intense accumulation of ^68^Ga-DOTA-WVP trace Col-IV expression in high-risk aneurysms and the early stages of dissection (22).

Col-IV can not only be exposed and posited on the intima beneath the TAAD, but also in the pathological tissues of renal fibrosis, atherosclerosis, and hepatic fibrosis, which is consistent with our results that the heart and kidneys also showed high uptake of ^68^Ga-DOTA-WVP (23). On one hand, the most reasonable explain of high accumulation of ^68^Ga-DOTA-WVP observed in heart and kidney in TAAD mice was caused of systematic aortic pathologies induced from BAPN administration in this TAAD model. But in the other hand, we should also pay an attention to the relationship of TAAD and heart as well as renal disease based on common pathogenesis. Renal injury was indicated by prolonged excretion time in mice with TAAD compared to that of the normal mice in the control group. However, previous studies demonstrated that type IV collagen that accumulates in the glomerular mesangium and renal interstitium contributes to the progression of chronic renal disease via the TGF-*β* signal path (24). Therefore, the importance of evaluation heart and renal abnormal function should be emphasized in patients with TAAD based on the common pathological change of type IV collagen deposition. Additionally, Kurata demonstrated that the severity of atherosclerosis and number of renal cysts were correlated with thoracic aortic circumference. Type IV collagen was noted in background renal tissue in cases with numerous renal cysts and suggests that a syndrome that affects the aorta and renal tubules, as well as atheroma, may exist (25). Therefore, abnormal exposure and deposition of Col- IV involving dysfunction of other important organs might be an target for noninvasive molecular imaging and nanosystem therapy for patients with TAAD. Whole-body PET/CT imaging and systematic evaluation using ^68^Ga-DOTA-WVP may play an important role in systematic assessing function changes of heart, renal and atherosclerosis in patients with TAAD. Reportedly, immune responses against collagen type IV contributed to vascular injury, affecting the development of atherosclerosis (26). Steffensen and Rasmussen summarized the important role causal involvement of collagen type IV in macrovascular diseases as the marker of intact basement membrane and stability of cellular homeostasis (27). The progression of atherosclerosis and other macrovascular diseases is accompanied by degrading collagen type IV and an increased production of abnormal interstitial collagen in the intima (28). Col-IV exposure is a prospective target to biological diagnosis and therapy and the application of ^68^Ga-DOTA-WVP PET/CT imaging targeted Col-IV detection in various other diseases accompanied by Col-IV deposition and exposure should be studied in future research.

Reportedly, the sST2 level was significantly elevated in patients with severe aortic valve stenosis who presented with pulmonary hypertension and associated with earlier death and high mortality (29). Another study demonstrated the prognostic value of sST2, which is an independent predictor of adverse outcomes in different aortic diseases (30). Additionally, sST2 can act as a heart damage biomarker because it acts as a decoy receptor for interleukin (IL)-33 and blocks the binding of IL−33 to membrane-bound ST2 to interrupt myocardial and vascular benefits (31). Our results showed that the level of sST2 was significantly increased in the ^68^Ga-DOTA-WVP PET/CT positive imaging group compared to the negative imaging group, which reflected the damage caused by sST2 in the heart and thoracic aortic wall. However, no relationship was observed between probe uptake and sST2 levels. CRP and D-dimer are systemic laboratory diagnostic biomarkers have been investigated and linked to the risk for aortic aneurysms or its outcomes but not sensitive and specific enough in clinical application. Reportedly, CRP is an independent risk factor in detection of vascular inflammation was associated with abdominal aortic aneurysm progression but few studies in TAAD (32). Inflammation and coagulopathy are non-specific characteristic in aortic aneurysm and dissection, while ^68^Ga-DOTA-WVP was designed to target imaging exposure of Col-Ⅳ to detect aorta damage earlier and specifically in unstable TAAD in this study. Therefore, no significant association between ^68^Ga-DOTA-WVP accumulation and the serum levels of these non-specific biomarkers in PET/CT positive and negative imaging groups of TAAD mice (33). The negative association also could be caused by insufficient sample size in this study and further research is needed on the topic.

The main limitation of this pilot study is the lack of reliable binding and inhibition experiments to quantify the quantitative-efficacy relationship between the uptake of ^68^Ga-DOTA-WVP and Col-IV expression, which will be studied further. Secondly, this pilot study lacks sufficient sample size for serial visualization of TAD progression using PET/CT to quantitatively analyze the relationship between probe uptake *in vivo* and Col-IV exposure at different time points as well as lack of confocal microscope photography scanning due to the COVID-19 pandemic. In our timeframe of experimentation, most aorta samples did not develop dissection. However, we observed that many animals with significant accumulation of ^68^Ga-DOTA-WVP in the aorta died from TAAD rupture. In our future study, we will continue analyzing the relationship between probe uptake and formation of dissection as well as the specificity of the probe for TAAD detection. Another limitation is that, although we showed that ^68^Ga-DOTA-WVP could be used to detect early TAD in a mouse model, this agent needs to be investigated further in large animals and eventually confirmed in humans. Furthermore, we should also visualize the aorta using CT-enhanced scans to co-locate TAAD lesions from structural information of size and functional imaging of Col-IV exposure via hybrid PET/CT imaging, which will be conducted in our following study.

## Conclusions

In summary, we prepared ^68^Ga-DOTA-WVP through a simple method with a high RCP and stability and demonstrated its ability to detect the biological characteristics of unstable aneurysms and early TAAD. Biological diagnosis of dissection plays an important role in clinical management. Thus, the current study proposes a promising method for dissection screening in high-risk patient populations, monitoring disease progression and assessing therapeutic response. Therefore, clinicians may benefit from PET-based whole-body risk assessments in guiding patient management and surgical decisions.

## Data Availability

The original contributions presented in the study are included in the article, further inquiries can be directed to the corresponding authors.
